# Effects of Nitrogen and Tensile Direction on Stress Corrosion Cracking Susceptibility of Ni-Free FeCrMnC-Based Duplex Stainless Steels

**DOI:** 10.3390/ma10030294

**Published:** 2017-03-15

**Authors:** Heon-Young Ha, Chang-Hoon Lee, Tae-Ho Lee, Sangshik Kim

**Affiliations:** 1 Ferrous Alloy Department, Korea Institute of Materials Science, Changwon 51508, Gyeongnam, Korea; lee1626@kims.re.kr (C.-H.L.); lth@kims.re.kr (T.-H.L.); 2 Department of Materials Engineering and Convergence Technology, Research Center for Aircraft Part Technology, Gyeongsang National University, Chinju 52828, Gyeongnam, Korea

**Keywords:** duplex stainless steel, nitrogen, stress corrosion cracking, pitting corrosion

## Abstract

Stress corrosion cracking (SCC) behavior of Ni-free duplex stainless steels containing N and C (Fe_balance_-19Cr-8Mn-0.25C-(0.03, 0.21)N, in wt %) was investigated by using a slow strain rate test (SSRT) in air and aqueous NaCl solution with different tensile directions, including parallel (longitudinal) and perpendicular (transverse) to the rolling direction. It was found that alloying N was effective in increasing the resistance to SCC, while it was higher along the longitudinal direction than the transverse direction. The SCC susceptibility of the two alloys was assessed based on the electrochemical resistance to pitting corrosion, the corrosion morphology, and the fractographic analysis.

## 1. Introduction

A variety of duplex stainless steels (DSSs) with approximately equal parts of austenite and ferrite in its annealed structure have been developed through alloying with 21 wt % to 29 wt % Cr, 1 wt % to 7 wt % Ni, and up to 4.5 wt % Mo and other elements such as Mn, W, and Cu balanced with Fe [1,2,3,4,5,6,7,8,9,10]. The DSSs have, in general, better resistance to localized corrosion and stress corrosion cracking (SCC) than single-phase austenitic or ferritic stainless steels [1,3,5,6,7,9,11]. The resistance to crack growth of DSS is also greater than that for single-phase stainless steel, because a crack in DSSs stops growing when it meets the other phase [1,5,6,10,11]. The DSSs are therefore widely used where both high corrosion resistance and excellent mechanical strength are required, such as for chemical tankers, desalination plants, chemical and petrochemical processes, pipelines, and oil and gas separators [1,5,6,8,9,11].

In order to accomplish desirable corrosion resistance and mechanical properties of stainless steels at low cost, a group of austenitic stainless steels with low Ni and high N and C contents (i.e., high interstitial alloy (HIA)) have recently been developed [12,13,14,15,16,17,18,19,20]. The interstitial alloying elements, N and C, are economical and effective austenite stabilizers, which can therefore substitute for the expensive austenite stabilizer Ni [12,13,17,18,19]. N and C are also known to increase mechanical strength without significant reduction in tensile ductility [12,13,14,18,20]. These elements in solid solution state are also reported to be effective at improving resistance to pitting corrosion [16,17,19]. The HIAs are therefore considered promising structural materials which can replace conventional FeCrNi-based austenitic stainless steels.

At present, the combined effect of alloying N and C has not been systematically investigated for DSSs. For this reason, FeCrMnC-based DSSs with different contents of N of 0.03 wt % and 0.21 wt % were designed and fabricated in the present study. The SCC susceptibility of these alloys was investigated by using a slow strain rate test (SSRT) in an aqueous NaCl solution, and the change in SCC susceptibility was discussed based on the electrochemical evaluation on the resistance to pitting corrosion, the corrosion morphology, and the fractographic analysis. The effect of loading direction (i.e., parallel and perpendicular to the rolling direction) on the SCC susceptibility of FeCrMnC-based DSSs was also studied.

## 2. Experimental Procedure

### 2.1. Materials

The specimen designation and chemical compositions of the investigated alloys are given in Table 1. The FeCrMnC-based DSSs were fabricated using a commercial pressurized induction melting furnace (VIM 4 III-P, ALD, Limbach-Oberfrohna, Germany) under N_2_ pressure of 1 bar. The ingots (10 kg) were homogenized at 1250 °C for 2 h and subsequently hot-rolled to 4 mm thick plates. In order to obtain the duplex microstructure with an equal volume fraction of austenite and ferrite phases, the hot-rolled plates of D1 and D2 alloys were solutionized at 1260–1280 °C for 1 h, and then quenched in water. For the micrographic observation, the specimens were polished using diamond suspension with a particle size of 1 µm and etched in a solution of 15 mL HCl and 85 mL ethanol for 45 s. The microstructures of the samples were observed using an optical microscope and scanning electron microscope (SEM, JSM-7100F, JEOL, Tokyo, Japan).

### 2.2. Corrosion Tests

The resistance to pitting corrosion of D1 and D2 alloys was evaluated by measuring pitting potential (E_pit_) and repassivation potential (E_rp_) through a cyclic potentiodynamic polarization test in a 2 M NaCl solution at 50 °C, at a potential sweep rate of 1 mV·s^−1^. For the polarization test, a three-electrode cell (a saturated calomel reference electrode (SCE), a Pt plate counter electrode, and a working electrode) connected to a potentiostat (Reference600, GAMRY, Warminster, PA, USA) was used. The test specimens were cold mounted in epoxy resin, and ground using SiC paper up to # 2000. Tested area (0.28 cm^2^) was controlled using electroplating tape. The cyclic polarization tests were performed on each specimen at least 5 times in order to confirm the reproducibility. 

**Figure 1:**
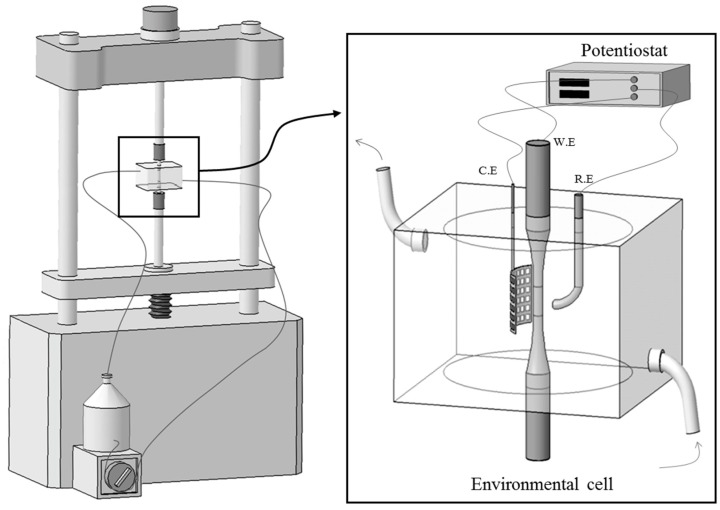
Schematic illustration of slow strain rate test (SSRT) set-up equipped with an electrochemical cell and a potentiostat.

**Figure 2:**
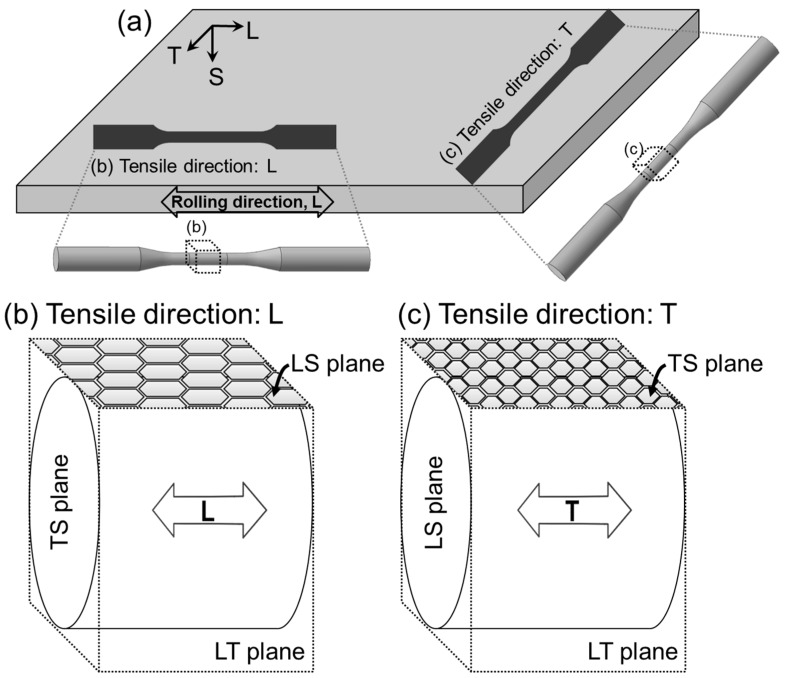
(**a**) Schematic diagrams of sample preparation. The samples were prepared (**b**) parallel to the rolling direction (longitudinal, L-direction); and (**c**) perpendicular to the rolling direction (transverse, T-direction).

SCC susceptibility of each specimen was evaluated using an SSRT method on a constant extension rate test machine (CERT-1, MTDI, Daejeon, Korea) equipped with an environmental three-electrode cell and a potentiostat (PAR VersaSTAT II, Princeton Applied Research, Oak Ridge, TN, USA), in accordance with ASTM G129. The detailed experimental set-up is presented in Figure 1. For the SSRT, the smooth tensile specimens with a gauge length of 24 mm and a diameter of 3.8 mm were prepared from 4 mm thick plate parallel to the rolling direction (longitudinal (L)-direction) and perpendicular to the rolling direction (transverse (T)-direction). The schematic diagram of the sample preparation is presented in Figure 2. 

Each specimen was ground using SiC paper up to # 1200, and encapsulated in the environmental cell shown in Figure 1. The environmental three-electrode cell consisted of a specimen as a working electrode, a platinum counter electrode, and an SCE reference electrode, positioned in a salt bridge. The SSRTs were conducted in the 2 M NaCl solution at 50 °C at a nominal strain rate of 10^−6^ s^−1^ under anodic applied potential of +0.05 V versus corrosion potential (E_corr_). The anodic applied potentials were determined based on the polarization test results obtained in the same condition. The solution was circulated by using a peristaltic pump at a speed of 20 mL·min^−1^. As reference data, the SSRTs were also performed on each specimen in laboratory air at the same strain rate of 10^−6^ s^−1^. The relative humidity for the laboratory air was controlled to be 45% to 55%. In addition, Vickers hardness of the alloys was measured using a micro-Vickers hardness testing machine (FM-700, Future Tech. Corp., Kawasaki, Japan) with a load of 1 kgf at 25 °C. The average hardness values were calculated from 10 results, excluding the minimum and maximum values, at different locations of each specimen surface.

The SCC susceptibility was then quantified by comparing the SSRT results obtained in air and aqueous chloride environments. After the SSRT, fracture surfaces and corrosion morphologies of tested specimens were observed by the SEM to understand the mode of fracture and the SCC mechanisms associated with initiation, propagation, and arrest of the crack formed on the specimen surface. The SCC susceptibility of the alloys were interpreted in correlation with the electrochemical behavior of the alloys. 

## 3. Results and Discussion

**Figure 3:**
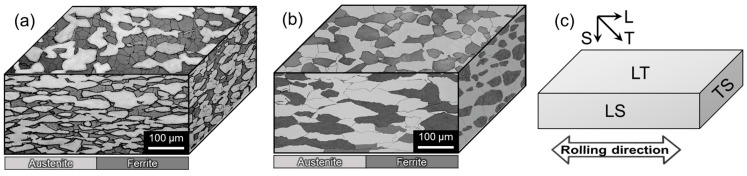
Three-dimensional optical micrographs of (**a**) D1 and (**b**) D2 alloys; (**c**) Definitions of the planes, LT, LS, and TS.

Figure 3a,b exhibit three-dimensional optical micrographs of D1 and D2 specimens, respectively, showing the austenite (shown as light phase) and ferrite (shown as dark phase). Cr-related precipitations such as Cr_2_N and Cr_23_C_6_ were not observed in the matrix, and nonmetallic inclusions such as MnO were rarely observed in both alloys with no notable difference between them. The average grain sizes of D1 and D2 specimens on the LT plane were 35 and 47 μm, respectively. The grains on the LS and TS planes were elongated along the rolling direction, and the grains were smaller on the TS plane than the LS plane. The image analysis indicated that the volume fractions between ferrite and austenite were approximately 50:50 for D1 specimen and 55:45 for D2 specimen.

**Figure 4:**
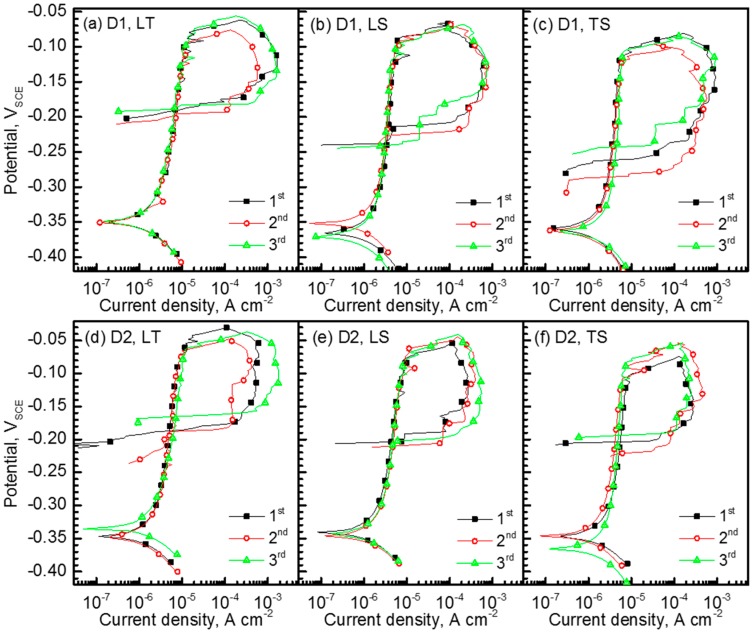
Cyclic polarization curves of (**a**–**c**) D1 and (**d**–**e**) D2 specimens measured in 2 M NaCl solution at 50 °C at a potential sweep rate of 1 mV·s^−1^. The polarization tests were conducted on (**a**,**d**) LT; (**b**,**e**) LS; and (**c**,**f**) TS planes.

**Figure 5:**
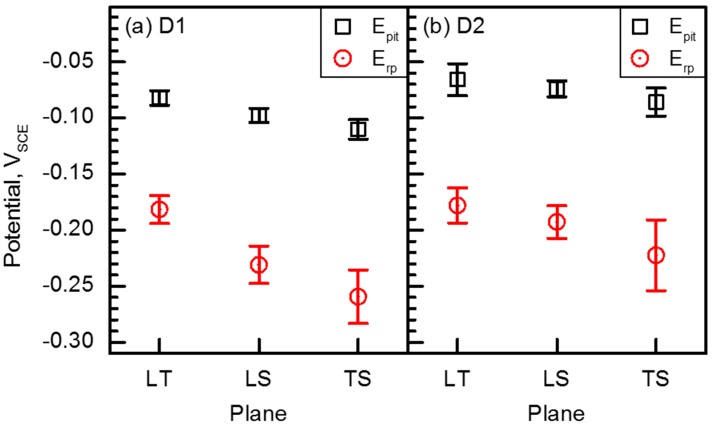
E_pit_ and E_rp_ values of (**a**) D1 and (**b**) D2 specimens measured on the LT, LS, and TS planes in 2 M NaCl solution at 50 °C at a potential sweep rate of 1 mV·s^−1^.

Figure 4 shows cyclic polarization curves of D1 and D2 specimens measured in 2 M NaCl solution at 50 °C at a potential sweep rate of 1 mV·s^−1^. The polarization tests were repetitively conducted on LT, LS, and TS planes at least 5 times on each plane, with the results showing good reproducibility. The polarization behaviors of D1 and D2 specimens were similar, exhibiting passive state at their E_corr_ levels (approximately −0.35 V_SCE_). From the polarization curves, it was possible to compare the resistance to pitting corrosion between D1 and D2 alloys. Average E_pit_ and E_rp_ values of both alloys were plotted in Figure 5. It was firstly noted that the resistance to stable pit initiation of D2 alloy was higher than that of D1 alloy. The E_pit_ values of D2 alloy measured on LT, LS, and TS planes were −0.066, −0.074, and −0.085 V_SCE_, respectively, and those of D1 alloy were −0.082, −0.098, −0.110 V_SCE_, respectively. In addition, the E_rp_ values of D2 alloy were also higher than those of D1 alloy. Secondly, Figure 5 showed that the E_pit_ and E_rp_ values depended on the plane orientation. The TS plane with elongated small grains exhibited the lowest E_pit_ and E_rp_ values for both alloys, while the highest E_pit_ and E_rp_ values were obtained on the LT plane. 

The higher E_pit_ and E_rp_ values indicate the higher resistance to stable pit initiation and greater tendency for repassivation (i.e., pitting extinction), respectively [21]. Thus, the polarization curves shown in Figure 4 suggest that the alloying N was beneficial to increase the resistance to stable pit initiation and repassivation tendency of FeCrMnC-based DSSs, and the highest pitting corrosion resistance could be obtained on the LT plane. As already known, N has beneficial effects on the pitting corrosion resistance of stainless steels. It is reported that the alloying N in the stainless steels increases the protectiveness of the passive film by forming an N-enriched layer at the interface between the metal and the passive film, and/or by increasing the Cr content in the film [22,23,24,25]. N can also prohibit the local acidification of the solution during active dissolution of the matrix (i.e., pH-buffering effect), accelerating repassivation process [24,25]. Regarding the plane orientations—because it is possible to exclude the influences of the precipitations and/or nonmetallic inclusions on the pitting corrosion resistance in the investigated alloys—it is reasonable to correlate the difference in the pitting corrosion resistance with the amount of grain (and/or phase) boundaries, which can provide the initiation site and the propagation path for pits for the D1 and D2 alloys. Figure 5 suggests that the higher resistance to pitting corrosion of LT plane was due to a relatively lower number of such sites and paths.

Based on the polarization curves in Figure 4, the potential value applied during the SSRT was determined to be +0.05 V versus E_corr_ value. The anodic polarization for the SSRT was required to exclude the influences of H and/or H_2_ on the SCC during the SSRT. In addition, the anodic applied potential should be lower than the E_rp_ value to minimize the influence of pure pitting corrosion on SCC. For this reason, the applied potential for the SSRT was determined as +0.05 V versus E_corr_ [19,26,27,28,29,30].

**Figure 6:**
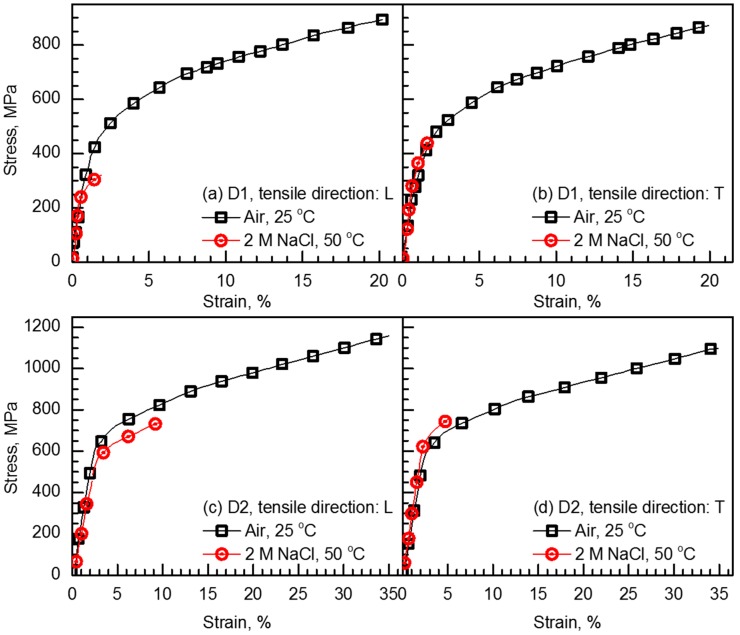
Stress-strain curves (strain rate = 10^−6^ s^−1^) of (**a**,**b**) D1 and (**c**,**d**) D2 alloys obtained in air at 25 °C and 2 M NaCl solution at 50 °C under anodic applied potential of +0.05 V versus E_corr_. The SSRTs were conducted along (**a**,**c**) L- and (**b**,**d**) T-directions.

Figure 6 shows the representative stress-strain curves of D1 and D2 specimens along L- and T-directions, obtained from the SSRTs at a strain rate of 10^−6^ s^−1^ in air at 25 °C and 2 M NaCl solution at 50 °C under anodic applied potential of +0.05 V versus E_corr_. Table 2 summarizes the SSRT results, the average values of which were obtained from duplicated test results. From Figure 6, it was noted that the tensile behavior of D1 and D2 specimens at slow strain rate was affected by the content of N, exposed environment, and tensile direction. Considering the tensile properties of D1 and D2 specimens in air, it was suggested that both tensile strength and ductility increased significantly with increasing N content. The yield strength and tensile strength values of D1 and D2 specimens along L-direction, for example, increased up to 50% and 35%, respectively, along with 87.9% improvement in tensile elongation and increasing N content from 0.03 wt % to 0.21 wt %. In addition, D1 and D2 specimens showed no notable anisotropy in tensile properties in air: the strengths and ductility for both L- and T-directions were similar to each other. The Vickers hardness values also confirmed the change in the mechanical properties by alloying N. On the LT, LS, and TS planes of D1 alloy, the average Vickers hardness values were 221.3, 248.6, and 241.6 Hv, respectively. For D2 alloy, the average Vickers hardness values were measured to be 288.1, 292.8, and 296.0 Hv on the LT, LS, and TS planes, respectively. It was clear that the alloying N increased hardness, and that the LT plane with the larger grain size than the two other planes exhibited slightly lower hardness value. 

The SSRT results showed that D1 and D2 alloys were susceptible to SCC in the 2 M NaCl solution at 50 °C under anodic polarization. As previously reported [19,31,32,33], the SCC susceptibility can be quantified by the reduction of tensile elongation (RTE) in SCC-causing environment with respect to that in inert environment (i.e., air in this study). The RTE along L-direction was, for example, 87.3% for D1 specimen and 64.3% for D2 specimen, indicating the reduced SCC susceptibility with increasing the N content. The yield strength and tensile strength values also decreased in 2 M NaCl solution at 50 °C under anodic polarization for both specimens. The reduction in strength was highly significant for D1 specimen, with the decrease of 11% to 64%, than for D2 specimen with the decrease of 0% to 33%. For D1 specimen, the SCC susceptibility with different tensile directions was not significant, but the RTE value of the SSRTed sample along T-direction was slightly higher than that of the sample along L-direction. In order to identify the effect of tensile loading direction on the SCC susceptibility of D1 alloy more clearly, the SSRTs were further conducted on D1 alloy along the tensile directions of L and T in mild SCC-causing environment of 0.6 M NaCl solution at 25 °C under applying potential of +0.05 V versus E_corr_. The RTE values of the D1 specimens SSRTed along L- and T-directions were 49.3% and 63.1%, respectively, in such a condition. Thus, it could be concluded that the D1 specimen SSRTed along T-direction exhibited higher SCC susceptibility than the one along L-direction. The dependence of the RTE value on the tensile direction was also observed in D2 specimen, such that the SCC susceptibility was considerably higher along T-direction than L-direction. 

**Figure 7:**
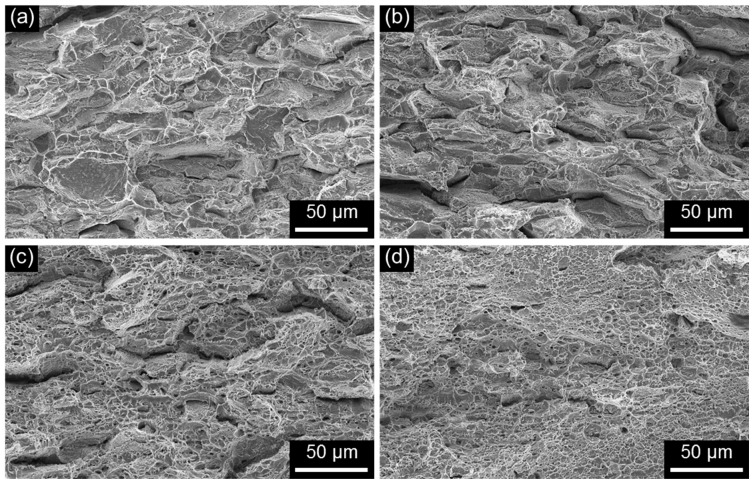
Scanning electron microscope (SEM) fractographs of (**a**,**b**) D1 and (**c**,**d**) D2 alloys after the SSRT conducted in air at 25 °C at a strain rate of 10^−6^ s^−1^. The SSRTs were conducted along (**a**,**c**) L- and (**b**,**d**) T-directions.

Investigating the fracture morphology, including the site of crack initiation (i.e., surface or internal) and the fracture mode (i.e., cleavage or intergranular cracking) of the SSRTed specimen, is important in understanding the SCC behavior. Figure 7 shows the SEM fractographs of D1 and D2 specimens SSRTed along L- and T-directions in air at a strain rate of 10^−6^ s^−1^. The combination of cleavage and dimpled rupture was observed in D1 specimens (Figure 7a,b), regardless of the tensile direction. The fracture morphology of each specimen correlated well with the microstructural features perpendicular to the tensile direction, such as the TS plane for L-direction and the LS plane for T-direction. The fracture surface of D1 specimen consisted of sharp facets on the ferrite and dimpled rupture on the austenite. The tensile fracture mode was significantly changed with increasing N content from the mixed mode (combination of cleavage and dimpled rupture) to almost 100% dimpled rupture mode. Unlike D1 specimen, D2 specimens showed dimpled rupture mode for both tensile directions (Figure 7c,d). The change in fracture mode well-explained the significant increase in tensile elongation with increasing N content. In addition, due to the microstructural features perpendicular to tensile direction, the secondary cracking along phase boundaries were frequently observed in D2 specimens with L-direction (Figure 7c). Apart from the secondary cracking, the characteristics of dimpled rupture mode appeared to be similar for both tensile directions. 

**Figure 8:**
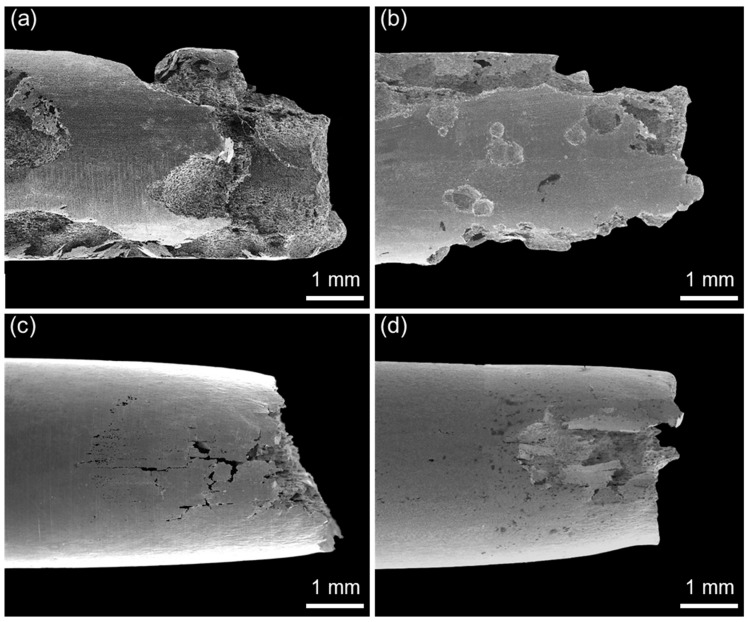
SEM micrographs of the side surfaces of (**a**,**b**) D1 and (**c**,**d**) D2 alloys after the SSRT conducted in 2 M NaCl solution at 50 °C under anodic applied potential of +0.05 V versus E_corr_ at a strain rate of 10^−6^ s^−1^. The SSRTs were conducted along (**a**,**c**) L- and (**b**,**d**) T-directions.

**Figure 9:**
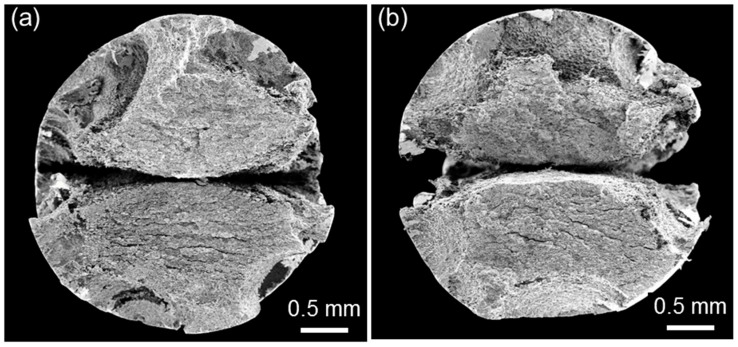

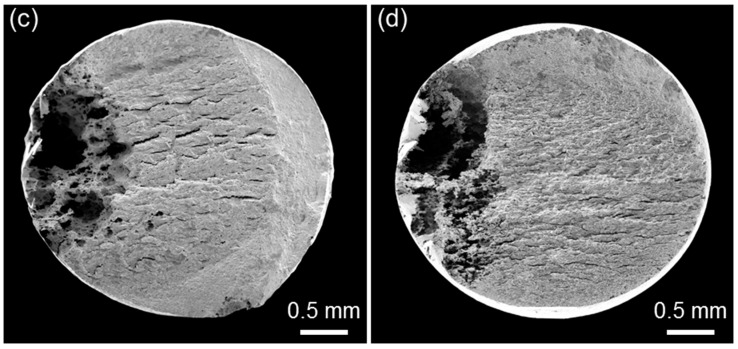
SEM micrographs of the fractured surfaces of (**a**,**b**) D1 and (**c**,**d**) D2 alloys after the SSRT conducted in 2 M NaCl solution at 50 °C under anodic applied potential of +0.05 V versus E_corr_ at a strain rate of 10^−6^ s^−1^. The SSRTs were conducted along (**a**,**c**) L- and (**b**,**d**) T-directions.

Figure 8 and Figure 9 show low-magnification SEM images of corrosion morphologies and fracture surfaces of D1 and D2 alloys, respectively, after the SSRT along L- and T-directions in the NaCl solution. It was found that the corrosion damage was significant in both specimens. Considering the three-dimensional microstructure of each specimen (Figure 2 and Figure 3), LT and LS planes were exposed to the environment for the specimens SSRTed along L-direction, while LT and TS planes were exposed for the specimen SSRTed along T-direction during straining in the aqueous NaCl solution. As the corrosion pits that formed on the exposed planes can act as initiation sites for SCC, the resistance to pitting corrosion of the exposed planes is important when considering the difference in SCC susceptibility, depending on the tensile orientation. As shown in Figure 8a,b and Figure 9a,b, a number of large corrosion pits were formed on D1 specimen surface, which grew into large-sized corrosion damage along the tensile direction for D1 specimen. Unlike D1 specimen, a few corrosion pits were observed on D2 specimen (Figure 8c,d and Figure 9c,d). The surface examination of SSRTed specimens clearly indicated that the formation of pits were suppressed with increasing N content in FeCrMnC-based DSSs, which matched well with the results from the polarization tests shown in Figure 4 and Figure 5. Such an improvement in pitting corrosion resistance was believed to be related to the enhanced stability of passive layer and pH-buffering effect of alloying N as demonstrated. In addition, Figure 8 and Figure 9 indicated that the tensile direction of L was more favorable than T-direction in reducing the corrosion attack during SSRT in NaCl solution. 

**Figure 10:**
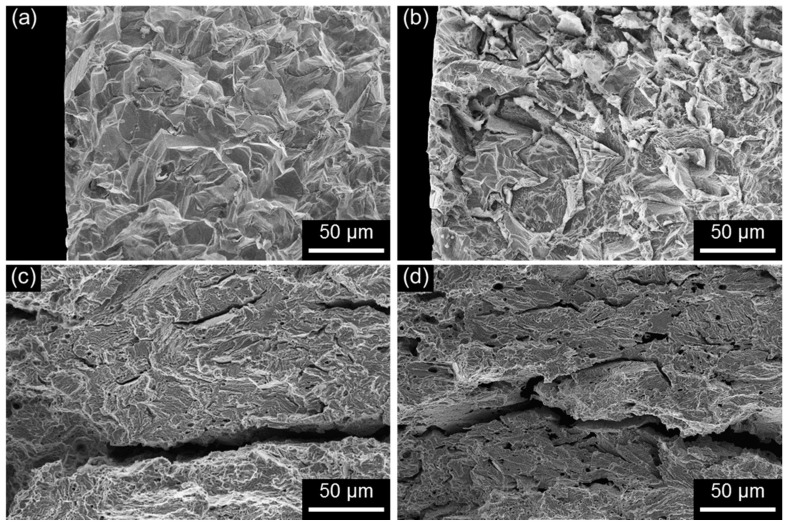
SEM micrographs of the crack initiation sites of (**a**,**b**) D1 and (**c**,**d**) D2 alloys after the SSRT conducted in 2 M NaCl solution at 50 °C under anodic applied potential of +0.05 V versus E_corr_ at a strain rate of 10^−6^ s^−1^. The SSRTs were conducted along (**a**,**c**) L- and (**b**,**d**) T-directions.

The change in fracture mode of FeCrMnC-based DSSs in response to the SCC-causing environment was clearly observed from the SEM fractographic analysis. Figure 10 shows the high-magnification SEM fractographs of D1 and D2 specimens, SSRTed along L- and T-directions in the 2 M NaCl solution at 50 °C under anodic polarization. The high-magnification SEM fractographs were taken at the crack initiation area. Comparison between those tested in air (Figure 7) and tested in the NaCl solution (Figure 10) confirmed that the tendency for cleavage fracture in the FeCrMnC-based DSSs was significantly promoted in the SCC-causing environment. It was particularly true for D1 specimen, showing almost 100% cleavage facet formed on both ferritic and austenitic phases (Figure 10a,b). Even though the mode of cleavage facet was also encouraged in D2 specimen in the NaCl solution (Figure 10c,d), the degree of cleavage was not as significant as that in D1 specimen. With respect to the tensile direction, both specimens showed no notable difference in the fracture mode in SCC-causing environment. 

**Figure 11:**
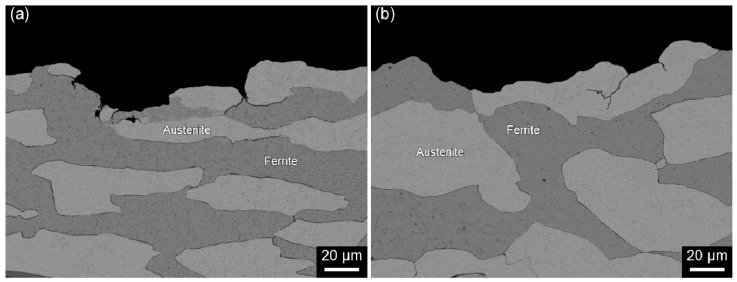
SEM micrographs of the cross-sectional view of (**a**) D1 and (**b**) D2 alloys after the SSRT conducted in 2 M NaCl solution at 50 °C under anodic applied potential of +0.05 V versus E_corr_ at a strain rate of 10^−6^ s^−1^.

However, there appeared to be some differences in the severity of secondary cracking and the size of facets due to the elongated nature of grain structure. Figure 11a,b shows the cross-sectional view of D1 and D2 specimens, respectively, SSRTed along L-direction in the NaCl solution. It appeared that the crack was initiated at the ferrite/austenite phase boundaries in D1 specimen. The cracks then propagated along the boundaries and appeared to be retarded by the presence of the austenitic phase (Figure 11a). For D2 specimen, the cracks propagated in transgranular manner across the austenitic phase (Figure 11b). Considering the micrographs in Figure 11 and the fractographs in Figure 10 together, it was believed that the cracks were propagated by the mechanism of cleavage for D1 specimen and quasi-cleavage for D2 specimen through ferritic and austenitic phases. The nature of SCC propagation mechanism, either cleavage for D1 specimen and quasi-cleavage for D2 specimen, is believed to be related to the size of grain and average aspect ratio of elongated grains. As demonstrated in Figure 3, relatively finer and less-elongated grain structure in D1 specimen provided more grain boundaries for intergranular cracking, while transgranular cracking was encouraged for D2 specimen with relatively greater and elongated grain structure.

The present SSRT results suggested that the alloying N was effective in increasing the SCC resistance of FeCrMnC-based DSSs. It was also found that SCC resistance of the investigated alloys was better along L-direction than T-direction. Combining the results from the polarization tests and SSRTs, it could be concluded that the enhanced SCC resistance by alloying N was due to the increase in the mechanical properties and resistance to pitting corrosion. In addition, it is considered that the difference in the SCC resistance with different tensile loading directions was primarily attributed to the resistance to pitting corrosion of the metallographic planes exposed to the environment during the SSRT; that is, LT and LS planes for the specimen SSRTed along L-direction, and LT and TS planes for the specimen along T-direction. As discussed in Figure 7, no notable anisotropy in tensile properties in air for D1 and D2 alloys were observed. As explained in Figure 10, no remarkable difference in the fracture mode was documented between TS and LS planes. Furthermore, secondary cracks were more frequently observed on the fractographs of SSRTed specimens documented on the TS plane than the LS plane. It was therefore believed that the better resistance to SCC of the SSRTed D1 and D2 specimens along L-direction than T-direction, despite the high possibility of the secondary cracking on the TS plane, could be associated with higher resistance to pitting corrosion of LS planes than TS planes, as shown in Figure 4. It has been well-established that the initiation and propagation of pitting has a strong relationship with the ease of SCC initiation and propagation [19,27,30,34]. The suppressed pit initiation and propagation during SSRT along L direction as compared to T direction largely increased the SCC resistance of FeCrMnC-based DSSs.

## 4. Conclusions

Ni-free DSSs containing N and C (Fe_balance_-19Cr-8Mn-0.25C-(0.03, 0.21)N, in wt %) were designed and fabricated, and the resistance to SCC of these alloys was evaluated by using SSRT in air and aqueous NaCl solution, with different tensile directions including parallel (longitudinal, L) and perpendicular (transverse, T) to the rolling direction. Based on the experimental results, the following conclusions were drawn.

(1) Alloying N was beneficial to resistance to pitting corrosion; it was also found that the TS plane with elongated small grains exhibited the lowest resistance to pitting corrosion, while the LT plane showed the highest resistance.
(2) The alloying N significantly improved both tensile strength and ductility of the FeCrMnC-based alloys. In addition, no notable anisotropy was observed in tensile properties in air; the strength and ductility for both L- and T-directions were similar to each other. The alloying N was effective in increasing the resistance to SCC, and higher SCC resistance was obtained when SSRTed along L-direction than T-direction.
(3) The enhanced SCC resistance by N addition was due to an increase in the resistance to pitting corrosion and the mechanical properties. The difference in the SCC resistance with different tensile loading directions was primarily attributed to the resistance to pitting corrosion of the metallographic planes exposed to the environment during SSRT.

## Figures and Tables

**Table 1:** Chemical compositions of the investigated alloys (in wt %).

Alloy	Fe	Cr	Mn	N	C
D1	Balance	19.151	7.889	0.034	0.278
D2	Balance	19.203	7.827	0.210	0.234
Si ≤ 0.15, Ni ≤ 0.01, S < 0.005, P < 0.003					

**Table 2:** SSRT conditions and the results for D1 and D2 specimens along L and T directions.

Alloy	Tensile Direction	Environment	Yield Strength MPa	Tensile Strength MPa	Elongation %	Reduction of Tensile Elongation %
D1	L	Air, 25 °C	420	858	15.8	
		2 M NaCl, 50 °C	287	308	2.0	87.3
	T	Air, 25 °C	413	888	16.3	
		2 M NaCl, 50 °C	366	449	1.8	89.0
D2	L	Air, 25 °C	631	1156	29.7	
		2 M NaCl, 50 °C	554	770	10.6	62.1
	T	Air, 25 °C	590	1094	30.8	
		2 M NaCl, 50 °C	595	767	5.5	82.1
